# The complete mitochondrial genome and phylogenetic analysis of sugarcane (*Saccharum* spp. hybrids) line 15a-53

**DOI:** 10.1080/23802359.2020.1820395

**Published:** 2020-09-21

**Authors:** Xiaolan Liu, Liping Xu, Youxiong Que, Hui Ling, Ze Yin, Ying Liu, Dinggang Zhou

**Affiliations:** a Hunan Key Laboratory of Economic Crops Genetic Improvement and Integrated Utilization, School of Life Science, Hunan University of Science and Technology, Xiangtan, PR China; b Key Laboratory of Sugarcane Biology and Genetic Breeding, Ministry of Agriculture/National Engineering Research Center for Sugarcane, Ministry of Science and Technology, Fujian Agriculture and Forestry University, Fuzhou, PR China; c Key Laboratory of Ecological Remediation and Safe Utilization of Heavy Metal-Polluted Soils, College of Hunan Province, Xiangtan, PR China; d Yulin Normal University, Agricultural College, Yulin, PR China

**Keywords:** Sugarcane, *Saccharum* spp. hybrid, mitogenome, phylogenetic tree

## Abstract

The complete mitogenome of *Saccharum* spp. hybrid 15a-53 was determined in this study, which contains two distinct circular chromosomes, Chromosome 1 and 2. The length of Chromosome 1 is 300,848 bp with the GC content of 43.93%, while Chromosome 2 is 144,713 bp in length with the GC content of 43.57%. In Chromosome 1, 7.14% of the genome (21,468 nucleotides) is coding DNA and 92.86% (279,380 nucleotides) are intergenic region, while in Chromosome 2, 8.20% of genome (11,865 nucleotides) are coding DNA and 91.80% (132,848 nucleotides) are intergenic region. Chromosome 1 contains 20 protein-coding genes (three *atp* genes, three *ccm* genes, two *cox* genes, one *mat* gene, one *mtt* gene, six *nad* genes, and four *rps* genes), and 21 non-coding genes (15 tRNA and six rRNAs), while in Chromosome 2, there are 13 protein-coding genes (four *nad* genes, three *rps* genes, two *atp* genes, one *ccm* gene, one *cob* gene, one *cox* gene, and one *rpl* gene) and five tRNA genes. Maximum Likelihood phylogenetic analysis indicated that 15a-53 is close to *S.* spp. hybrid ROC22, *S.* spp. hybrid FN15 and *S. officinarum* Khon Kaen 3. The complete mitochondrial genome herein will provide useful sequence information for phylogenetic and evolutionary studies for Saccharum and Poaceae.

Sugarcane (*Saccharum* spp. hybrids complex) is the most important commercial crop for the production of sucrose and ethanol in the worldwide (Garsmeur et al. 2018; Liu et al. 2020; Zhou et al. 2020). Modern commercial sugarcane varieties are all complex interspecies hybrids at least from the three species *S. spontaneum, S. robustum*, *and S. officinarum*, so the modern sugarcane cultivars exhibit an exceedingly complex interspecific aneupolyploid genome, so ‘*Saccharum* spp. hybrids’ is a general term for modern sugarcane (Garsmeur et al. 2018). Insect attack is a major issue in modern sugarcane cultivation. However, traditional cross-breeding is almost impossible in improving the insect resistance of sugarcane, due to lack of insect-resistant sugarcane germplasms and the characterization of highly complex polyploid–aneuploids with huge genome and large chromosome numbers of modern commercial sugarcane cultivars (Zhou et al. 2018). Genetic engineering provides an alternative (Zhou et al. 2018). *Saccharum* spp. hybrid 15a-53 is an insect-resistant transgenic *cry1Ac* line from receptor variety ROC22 (a *Saccharum* spp. hybrid from ROC5 × 69-463, the most widely cultivated sugarcane commercial variety during the past two decades in China) (Gao et al. 2016; Zhou et al. 2018), which could facilitate the development of insect-resistant sugarcane, and serves as germplasm for use in cross-breeding. The characterization of the complete mitogenome of sugarcane line *S.* spp. hybrid 15a-53 and its phylogenetic relationship within Poaceae were described in this study.

The complete mitochondrial genome of the sugarcane *S.* spp. hybrid 15a-53 was sequenced by Illumina Hiseq XTen and PacBio Sequel platform, assembled into the complete mitochondrial genome by SPAdes version 3.10.1 (Antipov et al. 2016), annotated by GeSeq (Tillich et al. 2017), and submitted to GenBank with the accession numbers of MT821853 (Chromosome 1) and MT821854 (Chromosome 2). The mitochondrial DNA (mtDNA) was extracted and purified from fresh yellowing seedlings of a single individual sugarcane line *S.* spp. hybrid 15a-53 (Chen et al. 2011), which from Fujian Agriculture and Forestry University, Fuzhou, Fujian Province (geographic coordinates: 26°9′8′′N, 119°24′24′′E), China. The specimen of 15a-53 was stored in the Key Laboratory of Sugarcane Biology and Genetic Breeding, Fujian Agriculture and Forestry University with store number 15a-53-FJ2016003.

The complete mitogenome of 15a-53 contains two distinct circular chromosomes, Chromosome 1 and 2. The Chromosome 1 is 300,848 bp in length with the GC content of 43.93%, and 7.14% of genome (21,468 nucleotides) are coding DNA while 92.86% of genome (279,380 nucleotides) are intergenic region. Chromosome 1 contains 20 PCGs (protein-coding genes, three *atp* genes, three *ccm* genes, two *cox* genes, one *mat* gene, one *mtt* gene, six *nad* genes and four *rps* genes), and 15 tRNA and six rRNAs non-coding genes. All these PCGs in Chromosome 1 use the initiation codon ATG except for *nad1* and *matR*, which begin with ACG and ATA, respectively. Regarding the stop codon of the PCGs in Chromosome 1, *matR*, *ccmC*, *ccmFn*, *mttB*, *nad6, nad7*, and *rps1* terminate with TAG; *atp4*, *atp8*, *cox1*, *cox2*, *nad1*, *nad9*, and *rps7* terminate with TAA; *atp1*, *ccmB*, *rps2* and *rps13* terminate with TGA, while *nad2* and *nad5* stop with CGG and GTA, respectively. The Chromosome 2 is 144,713 bp in length with the GC content of 43.57%, and 8.20% of genome (11,865 nucleotides) are coding DNA and 91.80% of genome (132,848 nucleotides) are intergenic region. Chromosome 2 contains 13 protein-coding genes (four *nad* genes, three *rps* genes, two *atp* genes, one *ccm* gene, one *cob* gene, one *cox* gene, and one *rpl* gene) and five tRNA genes. All the PCGs in Chromosome 2 start with ATG except for *nad2* and *nad5*, which begin with TTG and CCA, respectively, and *atp9*, *rps3* and *cob* stop with TAG, while *ccmFc*, *cox3, nad4* and *rps12* stop with TGA, and the other genes (*atp6*, *nad2, nad3, nad5*, *rps4* and *rpl16*) terminate with TAA.

The maximum likelihood phylogenetic tree with 1000 bootstrap replications was generated using PhyML version 3.0 (http://www.atgc-montpellier.fr/phyml/) based on the complete mitogenomes of sugarcane variety *S.* spp. hybrid 15a-53, eight other species from the family Poaceae, and two species from the family Cruciferae. GenBank accession numbers are as follows: *S.* spp. hybrid FN15(MT411890 and MT411891), *S.* spp. hybrid ROC22 (SRR11358604), *Sorghum bicolor* (NC_008360.1), *Zea mays* (NC_007982.1), *Oryza sativa* (NC_011033.1), *Triticum aestivum* (NC_037304.1), *Hordeum vulgare* (IBSC_v2.dna. Mt: 1:525599:1 REF), *S. officinarum* Khon Kaen 3 (LC107874.1 and LC107875.1), *Arabidopisis thaliana* (NC_037304.1), and *Brassica napus* (NC_008285.1). *A. thaliana* and *B. napus* were used as outgroups. The phylogenetic tree showed that *S.* spp. hybrid 15a-53 is very close to *S.* spp. hybrid ROC22, *S.* spp. hybrid FN15 and *S. officinarum* Khon Kaen 3 (Figure 1). The complete mitochondrial genome herein will provide useful sequences information for phylogenetic and evolutionary studies for Saccharum and Poaceae.

**Figure 1.**
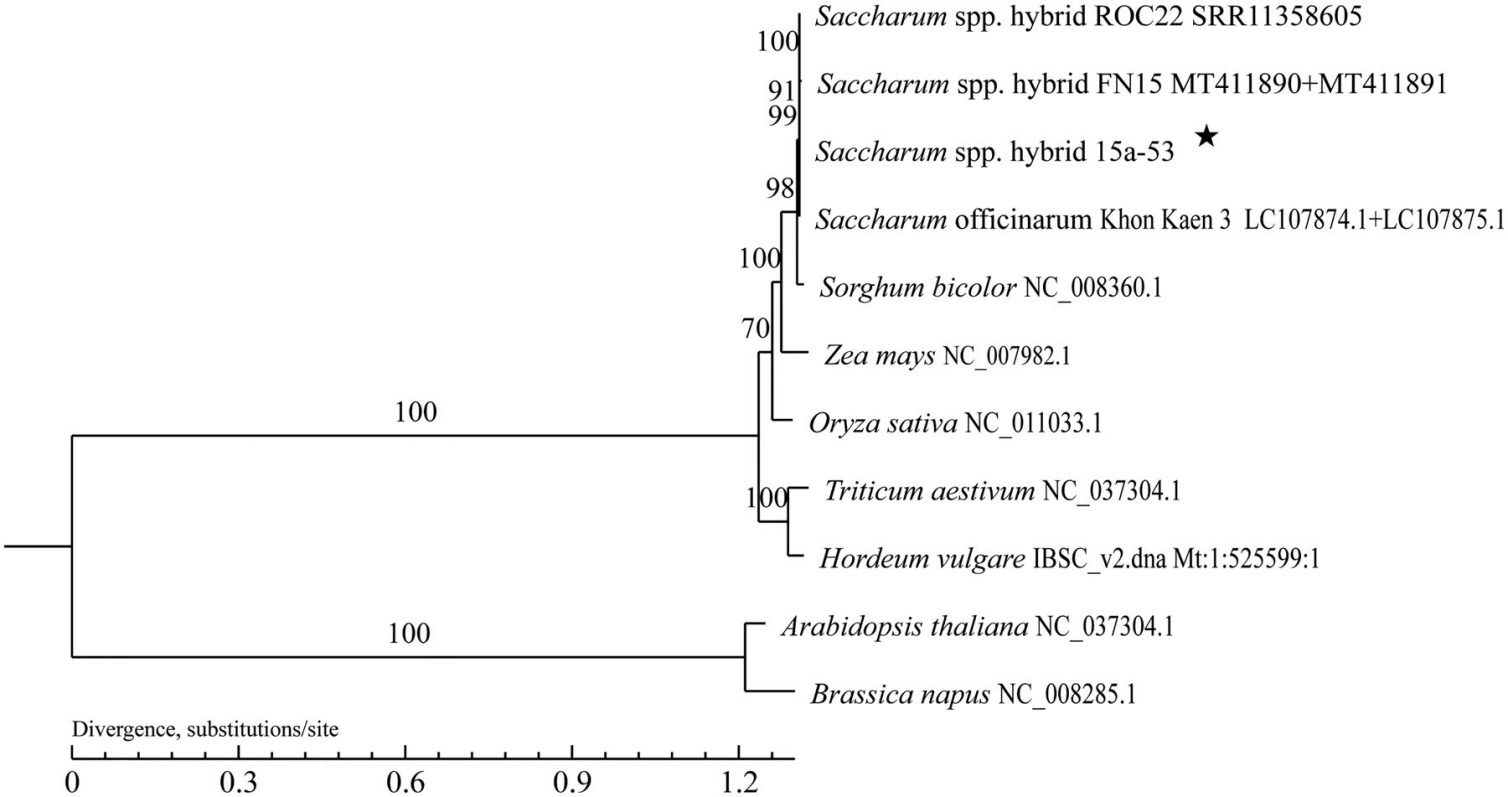
A maximum likelihood phylogenetic tree based on the comparison of mitochondrial genome sequences. GenBank accession numbers are listed after the species name. The numbers at the nodes are bootstrap percent probability values based on 1000 replications. The genome sequence in the present study is labeled with an asterisk.

## Data Availability

The data that support the findings of this study are available. The mitochondrial genome sequences rawdata of *S.* spp. hybrid 15a-53 were deposited in SRA with the accession number: SRR11358601 at the URL (https://www.ncbi.nlm.nih.gov/sra/?term=SRR11358601). The mitochondrial genome sequences of analyzed species (*S.* spp. hybrid FN15 and *S.* spp. hybrid ROC22; *S. bicolor*, *Z. mays*, *O. sativa*, *T. aestivum*, *S. officinarum* Khon Kaen 3, *A. thaliana*, and *B. napus*) were from the NCBI GenBank databases (the URL https://www.ncbi.nlm.nih.gov/genbank/) and the mitogenome of *H. vulgare* is available in Ensembl (http://asia.ensembl.org/index.html).
